# Associations between family factors and physical activity clustering in preschool children: a cross-sectional study

**DOI:** 10.3389/fpubh.2024.1367934

**Published:** 2024-10-31

**Authors:** Ting Huang, Guanggao Zhao, Jinmei Fu, Shunli Sun, Wendi Lv, Zihao He, Delong Chen, Ruiming Chen

**Affiliations:** ^1^School of Physical Education and Sport Science, Fujian Normal University, Fuzhou, China; ^2^School of Physical Education, Nanchang University, Nanchang, China; ^3^Jiangxi Sports Science and Medicine Center, Nanchang, China; ^4^College of Physical and Health, Jiangxi University of Traditional Chinese Medicine, Nanchang, China; ^5^School of Kinesiology, Beijing Sport University, Beijing, China

**Keywords:** physical activity, sedentary behavior, preschool children, family factors, clustering, parents

## Abstract

**Objective:**

This study aimed to examine the association between family factors and physical activity (PA) clustering in preschool children.

**Methods:**

Preschoolers’ PA and sedentary behavior (SB) were assessed consecutively for 7 days using ActiGraph accelerometers based on the cutoff counts developed by Pate et al. Information about children, their parents, and their families was collected using questionnaires. We developed a two-step approach to clustering PA both inside and outside of kindergarten. The Euclidean distance metric was utilized to distinguish between groups, while the Schwarz–Bayesian criterion was applied to identify the most optimal sub-group model. A one-way ANOVA was used to assess the clustering effect, and logistic regression was used to analyze the influencing factors of different clustering.

**Results:**

We collected data from 291 preschool children aged 3 to 6 years and divided them into three clusters—Inactive (50.2%), Active in kindergarten (26.8%), and Active outside kindergarten (23.0%)—with significant differences in PA and SB, revealing distinct temporal and spatial clustering patterns (silhouette coefficient = 0.3, *p* < 0.05). Furthermore, preschooler activity levels correlated significantly with factors including gender (OR = 0.35, 95% CI: 0.19–0.66), age (OR = 1.05, 95% CI: 1.00–1.10), birth weight (OR = 1.79, 95% CI: 1.16–2.76), paternal age (OR = 1.01, 95% CI: 1.00–1.02), and maternal income (OR = 0.68, 95% CI: 0.48–0.96).

**Conclusion:**

This study shows that the family environment or parents significantly influence the PA of preschool children. Older fathers may promote preschool children’s PA through greater educational focus and financial stability, while higher maternal income can provide more opportunities and resources for preschool children to engage in active lifestyles. Thus, it is suggested that families providing more attention and exercise opportunities for preschool children’s education can help improve their PA levels in the future.

## Introduction

Technological progress and the ease of transportation have brought plenty of convenience to human life but have decreased physical activity (PA) and increased sedentary behavior (SB) (1). This kind of trend in physical inactivity is recognized as “a global non-communicable disease prevalent in both developed and developing countries” (2). The World Health Organization (WHO) identifies it as the fourth leading risk factor for worldwide mortality (3). In addition, several studies have demonstrated that PA is essential for preschool-aged children’s health and mental wellbeing (4, 5). It significantly contributes to bone growth (6), the development of motor skills (7), and the building of self-esteem (8). The first 2000 days of life, from conception to age five, is when substantive learning about health-related behaviors occurs throughout life (9). Therefore, PA is essential throughout one’s life, and it is particularly crucial to foster it in preschool children whose habits are more malleable and receptive to change (10).

However, Lu (11) found that in Tianjin, China, preschool children engaged in only 6.6% of their day (both in and out of school) in moderate-to-vigorous physical activity (MVPA), and just 28% of them met the recommended levels of PA (32% of boys and 22% of girls). Currently, a significant portion of the population, including over 23% of adults and 80% of adolescents, are failing to achieve adequate levels of PA (1). Establishing healthy patterns of PA and SB from an early age is crucial for forming lifelong habits (10). Therefore, it is imperative to initiate interventions with preschool children to foster these habits and provide constructive guidance to enhance their health.

The pivotal role of family dynamics in shaping an individual’s health, particularly in the context of PA, has been underappreciated in the scientific discourse. In a decision tree analysis of preschoolers’ physical fitness, four factors affecting young children’s physical fitness are related to parents (12). The study of Watanabe advocates for public health interventions aimed at reducing childhood overweight/obesity by promoting measures such as lowering parental screen usage within families (13). Parents exhibit potential categorizations in their approach to children’s PA (14). This highlights a gap in the nuanced understanding of how family characteristics influence young children’s health through PA. The family environment profoundly influences children’s developmental trajectory, underscoring the critical importance of well-calibrated family-based interventions. However, the efficacy of previous studies has been hindered by a lack of detailed consideration of the diverse family factors of preschool children (15, 16). This oversight has led to interventions that are not sufficiently targeted, thereby reducing their potential to enhance preschoolers’ development significantly. Therefore, it becomes imperative to discern and address the family determinants that significantly influence the PA engagement of young children.

Cluster analysis is an unsupervised learning process used to find groupings/clusters of natural observations based on inherent characteristics within the data. It is an exploratory statistical method in statistics, widely used in medicine, biology, finance, and other fields. Some researchers have applied cluster analysis to identify behavioral patterns, physique, PA, dietary habits, etc. (17, 18). We hypothesize that preschool children’s PA performance has temporal and spatial distribution characteristics, and family factors influence PA performance differently. Therefore, this study aims to utilize a cross-sectional study to categorize the temporal and spatial characteristics of preschool children’s unsupervised PA performance and discuss the impact of diverse family factors on their PA levels. The findings in this study are expected to enhance the practical application of physical education in family settings for preschool children.

## Materials and methods

### Study design

**Figure 1:**
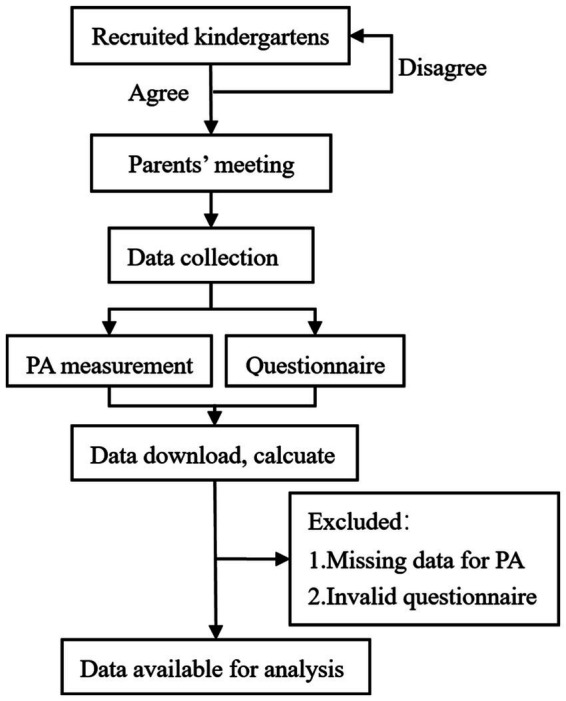
Flowchart of procedures for data acquisition of the study.

This cross-sectional study is a baseline data analysis from *The Physical Activity of Preschool Children Study* (Funding Number: 21BTY088). Recruitment was conducted through public welfare projects of Jiangxi Provincial Sports Bureau. The study’s leader negotiated with the kindergarten and obtained the consent of the kindergarten. Parent meetings are held with the support of the kindergarten. The benefits and associated risks of the study are carefully explained to the participant’s parents or legal guardians. A physical activity test was objectively measured using ActiGraph accelerometers, with the support of parents and kindergarten teachers. The questionnaires were distributed and collected with the assistance of Jiangxi Sports Science Medical Center, Nanchang Sports Bureau, and the kindergarten teachers. The study was approved by the Ethics Committee of FJNU (Fujian Normal University, ethics approval number: 19FJTK0051). Consent can be withdrawn from the study by participants and their parents or legal guardians at any time without explanation (Figure 1).

### Participants

A total of 330 preschool children were recruited from seven kindergartens in Nanchang, China, based on the regional economic development levels and the stratified sampling principle. All the parents/guardians of potential participants were fully informed of the study protocol and purpose during the parent–teacher meetings held in the seven kindergartens. The inclusion criteria for this study were (1) children aged 3 to 6 years old, (2) healthy and maintaining a typical lifestyle during the measurement period, (3) children without impairment, and (4) children’s parents or legal guardians provided informed consent. Finally, 330 children were eligible to participate in this research. According to the relevant data screening criteria, the movement behavior test data for 39 children were incomplete, or the questionnaire responses were deemed unqualified (incomplete answers, logical inconsistencies, or missing information). Therefore, the final group consisted of 291 children (boys, 57%).

### Physical activity

PA was measured over 7 consecutive days during waking hours using ActiGraph accelerometers (GT3X+, ActiGraph, Pensacola, FL, United States) placed on the right hip. Data collection was conducted at 15 s intervals, and PA was categorized into light physical activity (LPA) or MVPA based on the cutoff counts developed by Pate et al. for preschool children (19). LPA was delineated by 800 to 1,679 counts per minute (CPMs), whereas MVPA was characterized by counts equal to or exceeding 1,680 CPMs. SB corresponds to less than or equal to 799 (20). PA/SB inside the kindergarten refers to the period that children are engaged in activities while at kindergarten (e.g., from 9 am to 5 pm). PA/SB outside the kindergarten refers to the period before and after the school day. The rest day PA and SB refer to when children wake up until they sleep on rest days. Data from individual participants would be eligible for inclusion in the analyses if they have been recorded engaging in monitored PA for at least 3 days, including 1 weekend day, with each day accounting for a minimum of 8 h (21).

Before starting the data monitoring, the researchers, supported by the kindergarten principal, conducted a parent–teacher meeting to provide a comprehensive explanation of the study’s objectives and pertinent details to the participants’ parents. They were informed that the ActiGraph accelerometers should be worn continuously throughout the day, with the only exceptions being times designated for bathing, swimming (non-waterproofing), and sleeping. During the testing period, parental cooperation was needed for device placement and removal. All recruited parents signed the informed consent forms. After collecting data from the ActiGraph accelerometers, the data were analyzed with ActiLife (Version 6.13.3). For the preschool children whose data were missing or did not meet the requirements, the corresponding supplementary test was conducted with the consent of their parents. The researchers recorded children’s daily kindergarten attendance times and processed the time nodes in ActiLife to obtain PA and SB durations inside and outside kindergarten on weekdays and off days.

### Questionnaires

The parents of the participating preschool children were asked to complete a questionnaire to provide relevant information (Table 1). The questionnaire was designed based on the questions from studies by Zhao (22), which had been previously validated and tested for reliability. After distributing the questionnaire to the parents, they completed it and returned it the following day for collection by the research team.

**Table 1:** Specific content and variable assignment of the questionnaire.

First-level indicators	Second-level indicators	Variable types	Variable assignment
preschool children	Birth weight (kg)	Continuous	
	Birth length (cm)	Continuous	
	Months of age (m)	Continuous	
Parenting	Caregivers	Classification	1 = Parental care/2 = Grandparent care/3 = Parents and grandparents care together
	Daily sleep time (hour)	Continuous	
	Feeding patterns^a^	Classification	1 = Breastfeeding/2 = Artificial feeding/3 = Mixed feeding
	Whether to attend early education?^b^	Classification	1 = YES/2 = NO
Parents and family	Parental age (month)	Continuous	
	Parental BMI	Continuous	
	Parental education level	Classification	1 = Junior high school and less/2 = Vocational high school/technical secondary school/3 = High school/4 = Junior college/Higher vocational college/5 = College education
	Parental enjoys sports	Continuous	1 = Love/2 = A little love/3 = General/4 = Do not love/5 = Dislike
	Parental exercise frequency (day/week)	Continuous	1 = Everyday/2 = 5–6 times a week/3 = 3–4 times a week/4 = 1–2 times a week/5 = Never
	Parental occupation	Classification	1 = Managers/2 = Professionals/3 = Clerical support workers/4 = Services and sales workers/5 = Skilled agricultural, forestry, and fishery workers/6 = Plant and machine operators and assemblers/7 = Armed forces occupations/8 = Elementary occupations
	Parental income (CNY/month)	Classification	1 = ≤￥3000/2 = ￥3,001–￥5000/3 = ￥5,001–￥8000/4 = ￥8,001–￥10,000/5 = ￥10,001–￥15,000/6 = ≥￥15,001
	Marital status	Classification	1 = Married/2 = Unmarried^c^

To improve the accuracy of statistical analysis, the age of preschool children and parents in this study was calculated accurately to “month”.

a Feeding patterns from birth to 4 months of age.

b Early education is a “fitness center” or “interest class” for infants and young children that offers specialized courses, such as early education in music and early reading.

c Unmarried includes a single parent who has not remarried and the mother is raising the child, a single parent who has remarried, and the father is raising the child, a single parent who has remarried, and the mother is raising the child, and other conditions.

### Statistical analyses

IBM SPSS Statistics 25.0 software was utilized for statistical analysis. The cluster analysis encompassed six variables that evaluated the temporal and spatial aspects of preschool children’s PA, including PA and SB inside/outside the kindergarten (working day) and on rest day. A two-step cluster analysis used the Euclidean distance measure for group differentiation and the Schwartz–Bayes criterion to determine the optimal sub-group model. The two-step cluster analysis was deemed reliable for identifying the number of subgroups, the probability of individual classification into these subgroups, and the reproducibility of findings across clinical and various other datasets (23). In the pre-clustering phase, a sequential approach was used to preliminarily cluster cases by identifying dense regions within the attribute space under analysis. Subsequently, these preliminary clusters were statistically consolidated step by step until they formed a single comprehensive group (24).

Logistic regression was conducted to examine the impact of family factors, including continuous and categorical variables, on the clustering of preschool children. In logistic regression, different PA clusters were used as dependent variables, family factors were used as independent variables, and the reference category was set as Inactive. Continuous variables were presented as mean ± standard deviation and were evaluated using a one-way analysis of variance with the Tukey–Kramer *post-hoc* test. Normal distribution was assessed using the D’Agostino–Pearson test. A two-sided *p*-value of <0.05 was considered statistically significant in all analyses.

## Results

### Characteristics of preschool children

The characteristics of 291 participants (166 boys and 125 girls) who had complete data on PA and completed questionnaires by parents are shown in Table 2. The descriptive characteristics of preschool children’s various PA and SB are shown in Table 2. According to the PA measurement results, compared to girls, boys had significantly less SB than girls, while boys had substantially higher LPA, MVPA, and total physical activity (TPA) than girls.

**Table 2:** Characteristics of the analyzed sample.

Variables	Boys (*n* = 166)	Girls (*n* = 125)	Total (*n* = 291)	*p* for sex
Age (month)*	60.09 (7.8)	57.93 (7.2)	59.23 (7.4)	0.073
BMI (kg/m^2^)	16.37 ± 1.72	16.02 ± 1.24	16.22 ± 1.54	0.053
SB (min)	534.33 ± 66.89	550.77 ± 73.33	541.39 ± 70.08	**0.047**
LPA (min)	108.16 ± 23.58	101.90 ± 24.90	105.47 ± 24.31	**0.03**
MVPA (min)	76.67 ± 28.02	63.26 ± 24.81	70.91 ± 27.46	**0.000**
TPA/PA (min)	184.83 ± 48.00	165.17 ± 46.06	176.38 ± 48.09	**0.001**
SB in kindergarten (min/weekday)	266.87 ± 29.52	273.87 ± 28.13	269.88 ± 29.09	**0.042**
PA in kindergarten (min/weekday)	84.84 ± 29.89	77.66 ± 25.78	81.76 ± 27.19	**0.026**
SB outside the kindergarten (min/weekday)	253.52 ± 55.67	257.52 ± 52.85	255.24 ± 54.42	0.535
PA outside the kindergarten (min/weekday)	90.81 ± 27.83	82.80 ± 24.73	87.37 ± 26.79	**0.011**
SB (min/rest day)	549.30 ± 94.42	568.82 ± 95.72	557.68 ± 95.31	0.084
PA (min/rest day)	194.01 ± 64.60	170.27 ± 58.83	183.82 ± 63.19	**0.001**
Birth weight (kg)	3.41 ± 0.56	3.50 ± 0.87	3.45 ± 0.71	0.29
Birth length (cm)	50.78 ± 4.63	50.91 ± 3.78	50.84 ± 4.28	0.806
Caregivers				0.920
Independent parental care	36	24	60	
Independent grandparent care	4	4	8	
Parents/grandparents/babysitters care together	126	97	223	
Daily sleep time (hour)	10.27 ± 1.20	10.41 ± 1.43	10.33 ± 1.31	0.371
Feeding patterns				0.099
Breastfeeding	86	55	141	
Artificial feeding	48	36	84	
Mixed feeding	32	34	66	
Early education				0.748
Yes	23	19	42	
No	143	106	249	
Age of father (month)	434.72 ± 65.93	430.13 ± 69.83	432.75 ± 67.55	0.567
Age of mother (month)	407.39 ± 50.69	401.70 ± 53.15	404.94 ± 51.75	0.355
Father’s BMI (kg/m^2^)	23.61 ± 2.68	23.38 ± 3.11	23.51 ± 2.87	0.504
Mother’s BMI (kg/m^2^)	21.16 ± 2.29	20.87 ± 1.97	21.03 ± 2.16	0.272
Father’s education level				0.984
Junior high school and less	26	19	45	
Vocational high school/technical secondary school	37	31	68	
High school	47	33	80	
Junior college/Higher vocational	50	35	85	
College education	6	7	13	
Mother’s education level				0.937
Junior high school and less	27	19	46	
Vocational high school/technical secondary school	37	27	64	
High school	42	39	81	
Junior college/Higher vocational	56	34	90	
College education	4	6	10	
Father enjoys sports	2.66 ± 0.81	2.66 ± 0.75	2.66 ± 0.79	0.988
Mother enjoys sports	2.87 ± 0.67	2.90 ± 0.69	2.89 ± 0.68	0.705
Father exercise frequency	3.58 ± 1.11	3.78 ± 1.10	3.67 ± 1.11	0.119
Mother exercise frequency	3.84 ± 1.10	3.96 ± 1.10	3.89 ± 1.10	0.372
Father’s occupation				0.662
Managers	18	17	35	
Professionals	22	16	38	
Clerical support workers	14	7	21	
Services and sales workers	37	32	69	
Skilled agricultural, forestry, and fishery workers	3	1	4	
Plant and machine operators and assemblers	2	3	5	
Armed forces occupations	3	1	4	
Elementary occupations	67	48	115	
Mother’s occupation				0.894
Managers	19	9	28	
Professionals	14	16	30	
Clerical support workers	12	13	25	
Services and sales workers	49	32	81	
Skilled agricultural, forestry, and fishery workers	1	0	1	
Plant and machine operators and assemblers	0	1	1	
Armed forces occupations	1	0	1	
Elementary occupations	70	54	124	
Father’s income (CNY/month)				0.272
≤3,000	1	5	6	
3,001 ~ 5,000	2	3	5	
5,001 ~ 8,000	42	21	63	
8,001 ~ 10,000	95	59	154	
10,001 ~ 15,000	21	31	52	
≥15,001	5	6	11	
Mother’s income				0.591
≤3,000	24	19	43	
3,001 ~ 5,000	10	7	17	
5,001 ~ 8,000	69	45	114	
8,001 ~ 10,000	54	43	97	
10,001 ~ 15,000	6	9	15	
≥15,001	3	2	5	
Parental marital status				0.887
Married	162	123	285	
Unmarried	4	2	6	

*, median (interquartile range, IQR). Bold fonts indicate a *P* value less than 0.05, a factor with significant gender differences.

### Cluster analysis

**Figure 2:**
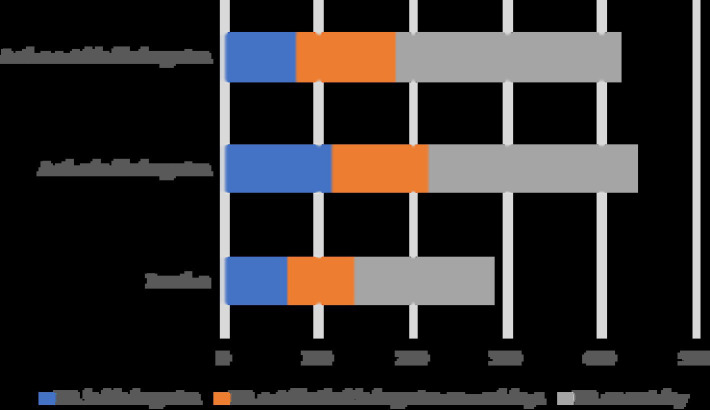
Bar graph of clustering.

The two-step cluster analysis reported a three-cluster classification as the optimal solution for the data considered in this study, with a silhouette coefficient of 0.3. There were significant differences in PA and SB among the three groups (*p* < 0.05), which showed good clustering results. Three clusters were identified and descriptively labeled according to their dominant features: (1) Inactive, (2) Active in kindergarten, and (3) Active outside kindergarten. The characteristics of these three clusters are shown in Figure 2 and Table 3.

Cluster 1: Inactive. This is the largest cluster, comprising 50.2% of participants (*n* = 146). It was characterized by significantly higher SB in kindergarten on weekdays, SB outside the kindergarten on weekdays, and SB on the rest days and less PA.
Cluster 2: Active in kindergarten. This second cluster comprised 26.8% of participants (*n* = 78) and was characterized by a high level of PA in kindergarten on weekdays and less SB.
Cluster 3: Active outside kindergarten. This smallest cluster comprised 23.0% of participants (*n* = 67) and was characterized by a high level of PA outside the kindergarten on weekdays and rest days.

**Table 3:** Descriptive of the different clusters.

	Inactive	Active in kindergarten	Active outside kindergarten
Sex: *n* (%)			
Boys	66 (45.2)	52 (66.7)	48 (71.6)
Girls	80 (54.8)	26 (33.3)	19 (28.4)
SB in kindergarten on weekdays	285.37 ± 18.61	232.95 ± 22.30*	279.08 ± 12.19*#
PA in kindergarten on weekdays	67.04 ± 17.56	114.28 ± 21.69*	75.95 ± 16.10*#
SB outside the kindergarten on weekdays	262.82 ± 52.06	237.14 ± 52.06*	259.78 ± 57.98#
PA outside the kindergarten on weekdays	71.01 ± 18.87	102.25 ± 26.03*	105.70 ± 19.65*
SB on the rest days	600.81 ± 81.27	496.04 ± 86.08*	535.48 ± 87.49*#
PA on the rest days	143.41 ± 40.77	216.69 ± 58.16*	233.58 ± 50.45*

*, *p* < 0.05 compared to the inactive cluster; #, *p* < 0.05 compared to the active in kindergarten cluster.

In contrast to the Inactive cluster, on weekdays in the kindergarten, children within the Active in kindergarten cluster exhibit a significantly higher level of PA, with an average difference of 52.42 min and a notably lower amount of SB, averaging a 47.24 min reduction. The Active outside kindergarten cluster also demonstrates a modest increase in PA, with an average difference of 8.91 min, and a slight decrease in SB, averaging 6.29 min less. Curiously, on weekdays outside the kindergarten, the PA levels of these two active clusters are comparable, with averages of 102.25 and 105.7 min, respectively, which substantially surpass the PA levels of the Inactive cluster. These findings suggest that the Active outside kindergarten cluster becomes more active once they return home, in contrast to their activity levels within the kindergarten environment. Conversely, the Active in kindergarten cluster is observed to be more active at kindergarten than at home.

On rest days, when all children are away from the kindergarten, both active clusters exhibit a pronounced increase in PA compared to the Inactive cluster, with average differences of 73.28 and 90.17 min, respectively. This underscores the fact that the Active outside kindergarten cluster stands out as the most active group when not in a kindergarten setting.

### Family factors

Logistic regression was carried out by taking the PA clusters as the grouping variable and the family factor as the independent variable. The results showed that the five elements were significantly correlated among different clusters (Table 4). Compared to the Inactive cluster, the activity factors of preschool children were related to gender, age, and birth weight. Specifically, gender emerged as a significant negative predictor, with a regression coefficient (B) of −1.05, indicating that girls are more likely to be categorized within the Inactive than boys, with an odds ratio (OR) of 0.35 and a 95% confidence interval (CI) ranging from 0.19 to 0.66, which suggests a diminished likelihood transitioning from the Inactive to Active in kindergarten and Active outside kindergarten for girls. The regression coefficient for age (Months of age) was positive (B = 0.05), indicating a gradual increase in the odds of shifting from Inactive to Active in kindergarten and Active outside kindergarten with each additional month of age, with an OR of 1.05 and a 95% CI from 1.00 to 1.10. Birth weight demonstrated a significant positive association, with a regression coefficient of 0.58, suggesting that higher birth weight is associated with an increased likelihood of belonging to Active in kindergarten and Active outside kindergarten rather than the Inactive one, with an OR of 1.79 and a 95% CI from 1.16 to 2.76. Although the effect of paternal age (Age of father) was less pronounced, the positive regression coefficient of 0.01 indicates a slight increase in the odds of moving away from the Inactive with each additional year of the father’s age, with an OR of 1.01 and a 95% CI from 1.00 to 1.02. Maternal income (Mother’s income) was negatively associated with the likelihood of being Inactive with a regression coefficient of −0.39, indicating that higher maternal income is associated with a decreased likelihood of the child being Inactive, with an OR of 0.68 and a 95% CI from 0.48 to 0.96. These findings elucidate the multifaceted determinants that influence an individual’s propensity for PA clustering and provide potential targets for interventions aimed at promoting active lifestyles.

**Table 4:** Logical regression results.

Factors	B	SE*	Wald	*p*	OR (95%CI)
Sex	−1.05	0.32	10.55	**0.000**	0.35 (0.19–0.66)
Months of age	0.05	0.02	4.02	**0.04**	1.05 (1.00–1.10)
Birth length	−0.03	0.04	0.62	0.43	0.97 (0.91–1.04)
Birth weight	0.58	0.22	6.93	**0.001**	1.79 (1.16–2.76)
Daily sleep time	0.15	0.12	1.46	0.23	1.16 (0.91–1.48)
Age of father	0.01	0.00	4.89	**0.03**	1.01 (1.00–1.02)
Age of mother	−0.01	0.01	1.51	0.22	0.99 (0.98–1.00)
Father’s BMI	−0.08	0.06	2.01	0.16	0.92 (0.83–1.03)
Mother’s BMI	−0.06	0.08	0.56	0.45	0.95 (0.82–1.10)
Feeding patterns	0.29	0.20	2.12	0.15	1.34 (0.90–1.97)
Caregivers	0.04	0.18	0.05	0.83	1.04 (0.73–1.48)
Early education	−0.58	0.47	1.50	0.22	0.56 (0.22–1.41)
Father exercise frequency	0.03	0.20	0.02	0.88	1.03 (0.70–1.52)
Mother exercise frequency	−0.11	0.19	0.35	0.55	0.89 (0.62–1.30)
Father enjoys sports	−0.25	0.24	1.04	0.31	0.78 (0.49–1.26)
Mother enjoys sports	0.01	0.27	0.00	0.96	1.02 (0.60–1.73)
Father’s education level	−0.11	0.22	0.26	0.61	0.90 (0.59–1.37)
Mother’s education level	0.19	0.24	0.63	0.43	1.21 (0.76–1.93)
Father’s income	0.01	0.19	0.00	0.98	1.01 (0.70–1.45)
Mother’s income	0.39	0.18	4.70	**0.03**	0.68 (0.48–0.96)
Father’s occupation	−0.01	0.07	0.01	0.92	0.99 (0.87–1.14)
Mother’s occupation	−0.05	0.08	0.50	0.48	0.95 (0.82–1.10)
Parental marital status	0.00	0.43	0.00	1.00	1.00 (0.43–2.31)

*, standard error. The bold font indicates that the *P* value is less than 0.05, indicating that the influence of this factor on the clustering of children’s physical activity is significant.

## Discussion

This study explored the relationship between preschool children with different PA characteristics and their family factors to provide a reference for the combination of their physical education and family education. We found significant differences in the clustering of PA inside and outside the kindergarten among the different clusters (*p* < 0.05), indicating a strong clustering effect. This suggests that preschool children’s PA exhibits clustering characteristics both temporally and spatially. Furthermore, in addition to the gender, age, and birth weight of preschoolers, in the family setting, the father’s age and the mother’s income may play some potential roles in the preschool children’s PA initiative.

Cluster analysis was adopted in this study, and it was found that cluster analysis could be applied to the natural observation grouping of PA in and outside the kindergarten with good results. The results showed that the silhouette coefficient of cluster analysis was 0.3, and there were significant differences among the groups. From a statistical perspective, clustering analysis and evaluation can adopt the average silhouette coefficient, which ranges from −1 to 1. The closer the coefficient is to 1, the better the clustering effect; conversely, the closer it is to −1, the worse the clustering effect (25). Therefore, the silhouette coefficient of this study is 0.3, which indicates that the clustering effect was good and reached a reasonable level. In addition, the clustering effect is not uniform from different professional perspectives. It is reasonable to summarize and explain, put forward a conceptual model of decision information, and express decision information according to data characteristics from a professional perspective to facilitate information analysis and processing (26). Therefore, this study achieves good clustering results from both statistical and professional fields and provides a basis for further policy decision-making, which includes implementing targeted family education, engaging healthcare providers, and tailoring interventions to different PA occurrence settings.

### PA performance and self-factors

According to our survey, boys were more active than girls. Similar to this study, previous studies have shown that boys were more active and participated in PA than girls (27). In addition, the PA of preschool children gradually increases with age, but the increase of boys was significantly more than that of girls (28). Studies showed that the emphasis on inclusion and description of gender differences was a scientific trend and a shift in research methodology due to differences in physiology, nutrition, and exercise metabolism between men and women (29). However, the preschool period is crucial for forming children’s gender consciousness (30). At approximately age 2, preschool children become aware of biological differences between boys and girls. By the age of 2–3, most preschool children can express their gender very clearly. At approximately age 4, preschool children’s gender awareness stabilizes, and they begin showing their “gender identity” through more apparent behaviors. Boys may be more likely to play with cars or soldiers fighting, while girls may be more likely to play with dolls or build houses. However, this more varied behavior may represent a difference in PA. Therefore, the results suggested that gender was also an essential factor in preschool, and future interventions and plans should be more targeted to compare the effects of different genders.

This study also showed a significant correlation between preschool children’s birth weight and PA clustering. However, there appears to be no direct causal relationship between the two. Studies have shown that gender, the record of preterm birth, and kindergarten can explain differences in average daily PA, intermediate school PA, and percentage of MVPA, with 44.7, 29.1, and 65.2% variation, respectively (31). The PA of pregnant women in the third trimester influences the baby’s birth weight (32). To some extent, both infants’ birth weight and PA performance are indirectly or directly related to mothers because infants’ birth weight is related to mothers’ nutrition and PA during pregnancy. In this study, the PA performance of preschool children acquiring different clusters was closely associated with parents and family environment, which may be the mediating factor of significant correlation between preschool children’s birth weight and different clusters.

These findings suggest that future interventions and policies should be designed with a gender-sensitive approach to promote PA among preschool children. This includes developing targeted programs, encouraging girls’ participation, and providing various activities catering to different interests. In addition, there is a need to enhance prenatal health education for mothers to improve birth outcomes, which can indirectly influence children’s PA levels.

### PA performance and family factors

This study showed that the father’s age and the mother’s income are important factors influencing children’s PA clusters. Preschoolers with older fathers tended to be more active, whether in or outside kindergarten, than preschoolers with younger fathers. Indeed, the effects were small but significant, with better neurodevelopment outcomes for children born to older parents (33). At the same time, other studies have shown that older fathers have an increased risk of having a child with low weight, intensive care unit admission, epilepsy, and musculoskeletal abnormalities (34, 35). Therefore, we need to view the results carefully. But, in general, older fathers, who tend to be more focused on their children’s education, are better off financially and more assertive, which can affect young children’s character development and healthy lifestyles. Although no studies have shown that the father’s age has a direct and significant relationship with the PA performance of young children, studies have shown that fathers’ sense of responsibility for family expenses is related to their participation in PA activities with their children (36). This involvement helps cultivate children’s active participation in these activities, thereby promoting their PA levels both inside and outside the kindergarten. This is one of the important reasons for the difference in PA between the clusters of “Active in kindergarten” and “Active outside kindergarten” compared to the “Inactive” cluster. It is worth noting that no significant disparity in activity levels was observed between the two Active clusters inside or outside the kindergarten. This could primarily be attributed to a father’s pivotal role in shaping a child’s character and habits. This influence is fundamental in fostering an active lifestyle and encouraging the development of a proactive personality in the child. Therefore, this study also highlights potential areas for future interventions to consider the father’s age of preschoolers.

According to the family system theory, PA and SB of family members influence each other (37). Previous studies showed that parents’ PA habits (15) and feeding styles (38) have varying degrees of influence on children’s PA. Parents’ attitudes, behaviors, and parenting styles profoundly impact children’s health behaviors, including PA (39). In this study, parents’ exercise behavior, liking for physical exercise, and feeding patterns did not significantly affect PA clustering. Previous studies have also shown that parents’ exercise behavior does not directly impact children’s physical health but may indirectly affect children’s behavior (PA) (40). Previous studies have focused more on children’s PA time rather than their specific performance in temporal and spatial tasks. This may explain why parents’ exercise behavior and attitudes did not have a significant influence in two key areas. One area was the indirect influence, with the mediating variables warranting further discussion. The other area, that this study focused on, was related to the temporal and spatial characteristics of children’s PAs, which may minimize the statistical gap between these factors, resulting in no significant impact.

Furthermore, this study also showed that the mother’s income was another factor affecting the PA clustering of children. The higher the mother’s income, the more time the child spends in PA, and the more likely the child is to be active, both “Active in kindergarten” and “Active outside kindergarten.” However, in Oda Malmo’s study, no significant correlation exists between parents’ or family income and MVPA (Activity level at leisure) (41). On the contrary, it is inconsistent with the results of this study. Malmo studied leisure time MVPA, while this study is the average daily total PA in, out of, or on rest days. This may be attributable to the differences in the intensity and type of PA.

In addition, the WHO states that preschool children need 60 min of MVPA daily and 180 min of TPA daily. This study pays more attention to the TPA of children, so there are differences in the duration of PA. In this study, the activity level during leisure can be understood as the time spent out of the park or on rest days. From the perspective of comprehensiveness, this study focuses on the whole PA of preschool children, including its temporal and spatial characteristics. Therefore, a new field has been opened in the total PA time of young children and PA performance in different environments, finding that young children’s PA is associated with maternal income. The results of the National Health and Examination Survey (NHAES) (2012) showed that family income was negatively correlated with the gross motor development of 3- to 5-year-old children (42). It can be explained that young children’s PA was a mediator between family income and physical health or athletic ability. Family income and socioeconomic status are directly related to parents’ parenting behavior and lifestyle, while PA and SB among family members influence each other. Therefore, the strong correlation between family income and athletic ability observed in the NHAES results implies a specific correlation between family income and preschool children’s PA.

Based on these analyses, the mother’s income is crucial in shaping the child’s PA initiative through economic means and lifestyle choices. This financial empowerment can significantly broaden the horizon of PA options for children, fostering an environment that encourages an active lifestyle. Conversely, a lower income might limit these opportunities as the family may face constraints in accessing facilities or affording the costs associated with organized sports or activities. This economic barrier could reduce the child’s exposure to diverse forms of PA, potentially leading to a more sedentary routine. In addition, the mother’s income can indicate the family’s socioeconomic status, which is known to influence health behaviors and outcomes. Families with higher incomes may have better access to information about the importance of PA and the means to prioritize and integrate it into their daily lives. This can create a cycle of health-conscious behavior that benefits the child’s PA levels. Therefore, higher maternal income significantly encouraged children in the “Active in kindergarten” and “Active outside kindergarten” clusters to be more active both in and out of kindergarten, compared to those in the “Inactive” cluster.

Parental and family factors are numerous and intertwined. However, if family environmental factors can be classified in a targeted manner to improve children’s PA performance, intervention measures for different aspects could be proposed. This approach would be constructive for improving children’s PA. Similarly, related studies on PA intervention in preschool children deserve long-term, in-depth discussion.

Thus, this study suggests that policy recommendations should consider the influential roles of paternal age and maternal income on preschool children’s PA levels. In response to these results, interventions can be more effective in encouraging positive habits in preschoolers, underscoring the importance of considering the family environment when developing strategies to improve children’s overall physical health. Interventions can be designed to support older fathers in actively engaging with their children, harnessing their potential, and fostering positive lifestyle and character development in their children. Training is also provided to young parents to enhance their knowledge and practical ability in PA parenting. In addition, this finding highlights the importance of maternal income, suggesting that higher income levels are associated with increased opportunities for children to engage in PA. These insights point to addressing socioeconomic disparities as an important issue, which in the case of sports is reflected in promoting equitable access to public sports resources. Future measures should consider expanding the input of public sports resources. Then, there should be a focus on the role of family and community support in creating an environment that fosters an active lifestyle from an early age. Similarly, training should be provided to community sports workers and parents to ensure that all children can participate in PA, which is both enjoyable and beneficial to their health and development.

### Strengths and limitations

To our knowledge, this study represents a pioneering effort to examine the temporal and spatial dynamics of preschool children’s PA and its intricate relationship with the family environment. This innovative approach provides a fresh perspective that could substantially enrich the research landscape. Despite its strengths—such as the novel research scope, a comprehensive analysis that blends quantitative and qualitative insights, and a broad sample base that bolsters the findings’ robustness—the study has its limitations. The regional bias of our sample may hinder the generalizability of our results, and the cross-sectional design limits our ability to establish causality. However, these limitations do not diminish the study’s potential to inform and inspire future interventions and policy development. The findings can guide the creation of targeted interventions sensitive to the specific contexts of the regions studied, thereby increasing the likelihood of their success. Moreover, the insights can inform policymakers to foster environments conducive to PA, advocating for accessible community facilities and family-oriented initiatives. Recognizing the limitations of a cross-sectional approach, future research should embrace longitudinal designs to elucidate the causal pathways and long-term impacts of family influences on children’s PA.

## Conclusion

In summary, this study demonstrated the associations between family factors and PA clustering in preschool children through a cross-sectional study. Three clusters were obtained with distinct differences in PA and SB levels, namely, “Inactive,” “Active in kindergarten,” and “Active outside kindergarten.” The results indicated that the father’s age and the mother’s income are noteworthy in the temporal and spatial manifestation of PA in preschool children. This suggested that families could promote preschool children’s PA through greater educational focus and financial stability and provide more opportunities and resources for preschool children to engage in active lifestyles. This study underscores the significant influence of the family environment and parental behaviors on the PA of preschool children. By innovatively examining the temporal and spatial dimensions of family impact, the research opens avenues for future studies to implement and assess additional interventions. These interventions could include guiding parents to focus more on their children’s physical education and could offer more opportunities and platforms for children to engage in physical activities, thereby fostering an environment conducive to enhancing their PA levels.

## Data Availability

The original contributions presented in the study are included in the article/supplementary material, further inquiries can be directed to the corresponding author.
